# Knitting for heart valve tissue engineering

**DOI:** 10.21542/gcsp.2016.31

**Published:** 2016-09-30

**Authors:** Albert Liberski, Nadia Ayad, Dorota Wojciechowska, Dorota Zielińska, Marcin H. Struszczyk, Najma Latif, Magdi Yacoub

**Affiliations:** 1 Sidra Medical and Research Center, P.O. Box 26999, Doha, Qatar; 2 Mechanical Engineering and Material Science Department, Military Institute of Engineering (IME), Rio de Janeiro, RJ, Brazil; 3 Lodz University of Technology, Faculty of Material Technologies and Textile Design, Department of Material and Commodity Sciences and Textile Metrology, ul. Zeromskiego 116, 90-924, Lodz, Poland; 4 Institute of Security Technologies “Moratex” 3 M, Skłodowskiej-Curie Street 90-505 Lodz, Poland; 5 Imperial College of Science and Technology, London, UK

## Abstract

Knitting is a versatile technology which offers a large portfolio of products and solutions of interest in heart valve (HV) tissue engineering (TE). One of the main advantages of knitting is its ability to construct complex shapes and structures by precisely assembling the yarns in the desired position. With this in mind, knitting could be employed to construct a HV scaffold that closely resembles the authentic valve. This has the potential to reproduce the anisotropic structure that is characteristic of the heart valve with the yarns, in particular the 3-layered architecture of the leaflets. These yarns can provide oriented growth of cells lengthwise and consequently enable the deposition of extracellular matrix (ECM) proteins in an oriented manner. This technique, therefore, has a potential to provide a functional knitted scaffold, but to achieve that textile engineers need to gain a basic understanding of structural and mechanical aspects of the heart valve and in addition, tissue engineers must acquire the knowledge of tools and capacities that are essential in knitting technology. The aim of this review is to provide a platform to consolidate these two fields as well as to enable an efficient communication and cooperation among these two research areas.

## General characteristic of aortic heart valve and concept of smart scaffold

The aortic heart valve (AHV) is responsible for the unidirectional flow of blood out of the left ventricle, preservation of myocardial function and control of the coronary blood flow^1^. It closes and opens over 100,000 times a day and will function normally over the lifetime of a human being^2^. During this period, it is exposed to shear stress, bending forces, as well as strain and loading forces that are a result of its hemodynamic environment.

**Figure 1.**
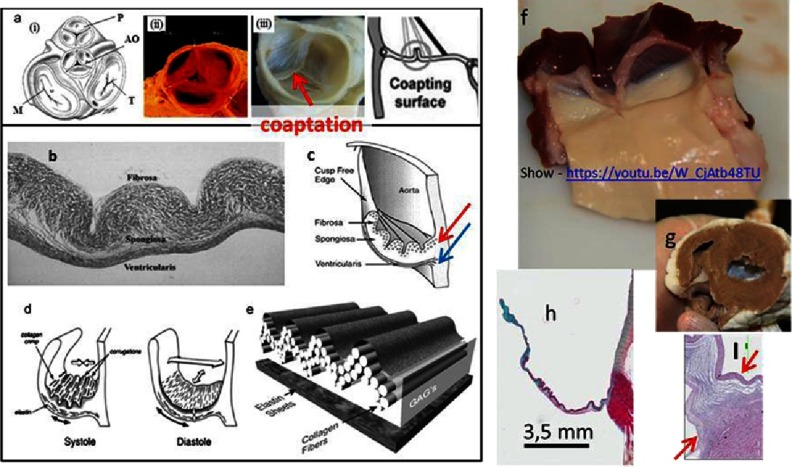
The basic structural/functional aspects of the heart valve (HV). Schematics of the 2D position of the four valves on valvular basal plane of the heart where P: pulmonary valve, AO: aortic valve, M: mitral valve, and T: tricuspid valve (a-i), porcine pulmonary heart valve (a-ii), decellularized porcine aortic heart valve (a-iii). The leaflet consists of three distinguishable layers: fibrosa, spongiosa and ventricularis (b). Each layer contains specifically oriented fibers, red arrow indicates circumferentially oriented fibers of collagen, blue arrow indicates radially oriented fibers of elastin (c).The types (collagen, fibronectin, elastin and others) and arrangement of fibers are responsible for the function of the valve (opening & closing) (d, e). The fibrosa layer is a continuation of aorta and ventricularis is a continuation of the ventricular chamber. (f) The view of sheep aortic valve from ventricular side, after dissecting the heart (g). The leaflets contain glycosaminoglycans (GAGs, mainly in spongiosa, blue color), which works as a lubricant between layers and a shock absorber (h). The hinge area (in-between the arrows) histology highlights the importance of fiber arrangement (I). The cells are housed and maintained in a highly organized network of fibers. Reprinted with permission from^4^.

As a living, dynamic organ, it can adapt to its complex biomechanical and mechanical environment through passive and active communication between its constituent parts (see Figure 1)^3^. An example of passive communication is the influence of the shapes of the sinuses on forming the vortices that are important for valve closure and for maintaining coronary flow during systole. Active communication occurs when structures in each part of the valve (cusp, annulus, sinus, sinotubular junction) change shape, size and stiffness during specific parts of the cardiac cycle^1^. Such changes guarantee optimal ejection of blood from the left ventricle, valve opening and closure, adequate coronary perfusion as well as cooptation of the leaflets to prevent blood backflow into the left ventricle. This highlights the importance of using tissue engineering to develop heart valve substitutes that contain living cells and have the ability to assume the complex functions of the native valve.

The ideal smart scaffold should be non-immunogenic, resorbable and capable of attracting, housing and instructing cells to produce a particular phenotype^5^. It should also reproduce the performance and mechanical properties of the native valve, both in the short and long term.

Therefore, the smart scaffold is and for some time will be, the best option for developing a clinically relevant Tissue Engineered Heart Valve (TEHV) as it enables the remodeling of the construct with the patients’ cells and decreases greatly the possibility of a detrimental immune response^5,6^.

The main types of HV scaffolds currently being considered can be divided in two categories, namely, biological scaffolds and scaffolds based on synthetic polymers. The first category includes decellularised valves and whole organs^7^, decellularised non cross linked ECM^6^, amniotic membrane^8^ or scaffolds made out of alginate manufactured from sea weed^9^. The second type of intelligent scaffolds are made out of synthetic polymers^10^, including polyglycolic acid (PGA)^11^, poly (L-lactic acid) (PLL)^12^, copolymer blend PLGA^13^, Poly-ξ-caprolactone (PCL) and many others^14^. These synthetic polymers can also be enhanced with antibodies^15,16^, peptides^17,18^, aptamers^19^ and enzymes^20^ in a way that enables the scaffold to attract and instruct host cells, either from blood or surrounding tissue and induce them to differentiate to a tissue specific phenotype.

**Figure 2.**
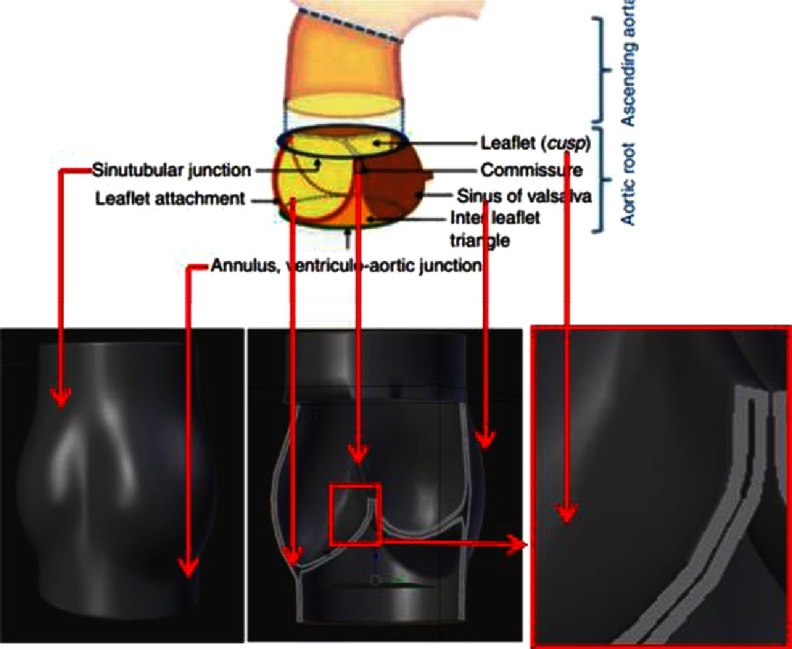
Geometry of the native (top panel) HV, and intelligent scaffold (bottom panel). Top panel was reprinted with permission from^2^.

Using current state-of-the-art technology, it is most reasonable to focus on combining “the best elements” of the available strategies by constructing a textile-based scaffold with the required functionality and characteristics (Figure 2). The logic behind such an application of textile technologies is to utilize its advantages that have already been shown in the wide use of textile medical grafts in clinical practice. One major advantage of textile techniques is their utility in reproducing the anisotropic properties of the valve^21,22^.

## Basics of knitting and its applications

Knitting is the process of manufacturing fabrics of interlooped yarns using hooked needles. The basic elements of the knitted fabrics are the loops. The loops can be closely or loosely constructed allowing them to be stretched in any direction, even if the yarn itself has low elasticity^23^.

**Figure 3.**
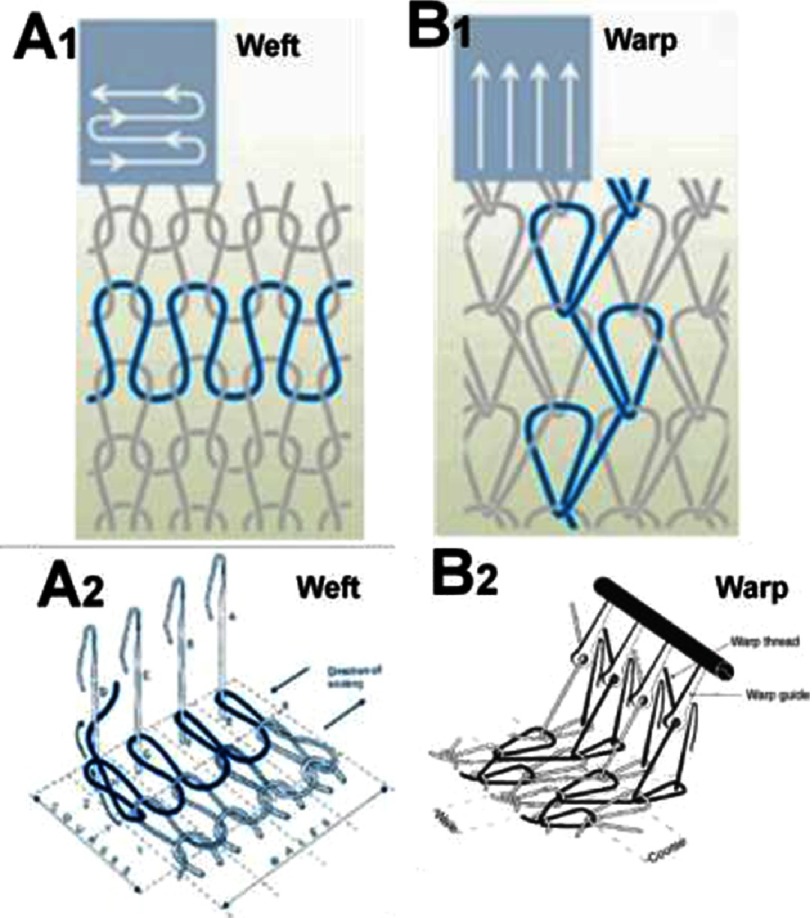
Basic patterns of weft (A1) and warp (B1) knitting. Horizontally aligned yarns, fed crosswise along the fabric (A2). Parallel yarns lengthwise zigzag along the fabric, each loop securing a loop of a neighboring strand from the previous row (B2). Courses and wales are indicated on both A2 and B2. A1 and B1 were reprinted with permission from^24^, and B2 was reprinted with permission from^23^.

The fabric can be characterized by the number of courses and wales per cm^2^. *Course* is a line of loops in the horizontal direction and *wale* is a line of loops in the vertical (Figure 3A2 and 3B2). A knitted fabric having more courses will be rigid and stable in length while a fabric with more wales will be rigid and stable in width.

There are two basic systems of knitting; the weft and warp knitting (Figure 3). In weft knitting, the yarns come from the horizontal direction, while in warp knitting the yarns come from the vertical direction. Each horizontal course in weft and vertical in warp knitting can be individually chosen (see Figures 3 and 5).

It is worth noting that weft knitting is useful for generating coherent objects stretchable in all directions with various choices of pattern, while warp knitting is more suitable for open net-like, branched structures with programmable directional elasticity. Therefore, for HVTE, warp knitting may be the technique of choice for leaflets, while weft knitting may be more useful to make a scaffold for sinus of Valsalva (see Figure 2). Additionally, because the nature of warp-knitted loops is more knot-like, the warp-knitted fabrics are more resistant to ripping. This particular aspect makes them more usable for medical textiles applications.

**Figure 4.**
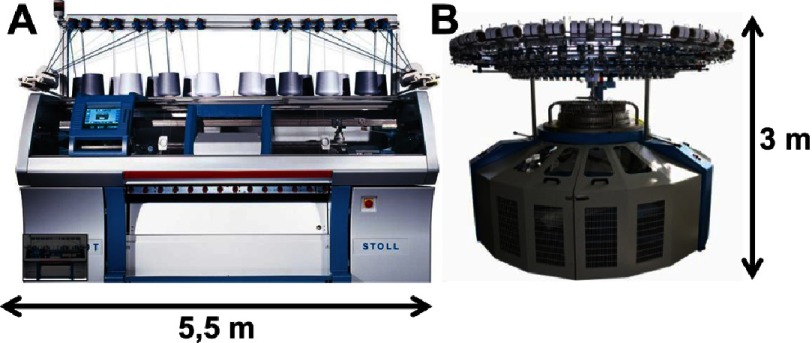
Examples of automated knitters. Flat-bed (A) and circular (B) knitting machines. Reprinted with permission from^25,26^.

Usually, the weft knitting is performed by the so-called circular knitter machines, while warp knitting by flat knitting machines (Figure 4). Both types of machines are capable of fabricating tubular and even seamless garments. This is interesting from the point of view of HVTE, as the aortic root is a tubular object with a complex geometry (sinuses and leaflets, see Figures 1 and 2).

**Figure 5.**
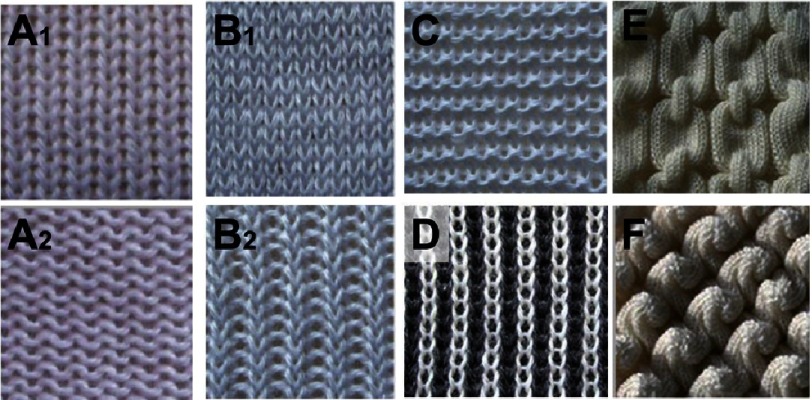
The popular types of weft knitting. Single jersey, face (A1) and back side (A2). Purl stitch, face (B1) and back side (B2). Rib stitch (C) and interlock stiches (D). Examples of links-links patterns (E) and (F). Reprinted with permission from^31^.

The most prevalent method of knitting 3D garments is to knit the bi-dimensional fabrics first followed by post-processing, e.g. by stitching to form the final geometry of the product. Currently, there is a strong trend in the direction of seamless garments (Figure 9)^27^. For that purpose, both weft and warp knitting could be used, however the advantage of warp knitting is that it can deliver hosiery (Figure 9E) or jackets with sleeves in one run while in weft knitting, sleeves and legs need to be attached separately.

Indeed, the seaming process makes fabrication more difficult to produce and more expensive. Nevertheless, the seaming methods are currently very precise and efficient, hence the necessity of seaming does not disqualify weft-knitting as a method to deliver complex 3D geometries such as a HV scaffold. Therefore, a compromise between complexity of process and required geometry of design should be made.

In principle, when using unconventional yarns (bioresorbable polymers for example) for fast prototyping, weft knitting is more advantageous since it requires, in its basic setting, only one cone of yarn, while warp knitting demands that each needle needs to have its own yarn. Thus, for warp knitting, a large number of cones/bobbins must be delivered to the knitting facility. As an alternative, the cone could be split, but this is a costly and a technologically advanced operation.

Table 1 outlines a summary of the differences between warp and weft knitting as well as the importance of the characteristics of each process for HVTE.

**Table 1:** Comparison of weft and warp knitting processes^24^ with comments on HVTE relevance.

Weft knitting	Warp knitting	HVTE relevance
Stretches in both directions	Stretches mainly width wise	This characteristic of dimensionally-specific stretch is critical for mimicking the anisotropic mechanical properties of specific layers of leaflets.
Yarns can be supplied from single or few cones	Yarns must be provided from the warp beam	Warp-beam preparation is an expensive, time-consuming process and increases the “dead volume” of yarns (amount that needs to be loaded to machine prior obtaining the fabric. Weft knitting is advantageous for knitting with expensive yarns as it allows reducing their usage.
Possible to knit with one yarn	Needs multiple warp yarns	Preparing the warp beam requires a large number of cones. Alternatively, the cone could be split. Splitting is a costly technological operation.
Stable-fibre yarn can also be processed	Only strong filament yarns can be processed	Most biopolymeric yarns are in the form of filament; nevertheless, some may be disqualified for warp knitting due to insufficient strength.
Less versatility	More versatility	For quick prototyping versatility may be less important than for final optimal design of HV scaffold. Noteworthy, 3D knitting (vital for HVTE) is done with weft knitting. The higher versatility of warp knitting relates here to available pattern rather than to shapes.
Loops are not uniform	Loops are uniform	As long as variations in sizes of loop do not critically affect the properties of construct overall, both methods of knitting can be applied for fabrication of the HV scaffold.
Dimensionally less stable	Dimensionally more stable	Dimensional stability is critical for preserving the designed geometry of the construct.
Less expensive equipment	Expensive equipment	For R&D, the price of the equipment is a critical factor that needs to considered, especially if knitting services are outsourced. The machine owners will hesitate to test atypical/unknown yarns on expensive tools. It will be easier for them to accept the risk if circular knitting is performed to ensure yarn suitability for processing.
Low running cost	High running cost	Expensive machines need to run continuously to pay back to the owner. It is easier to prototype with a low-cost machine.
Short production runs	Mass production	Short production runs are more suitable for prototyping phases of the project.
Less space required	More space required	For a laboratory environment, a compact equipment is usually preferred.

Detailed classifications of knitting are presented elsewhere^23,28,29^ and only a few examples are shown on Figures 3 and 5. The most popular types of warp knitting include tricot, Raschel (see Figure 4) and stitch-bonding and for weft, these are single jersey, purl, rib and interlock types of stitches (see Figure 5)^23^.

From the perspective of HV scaffold construction, a very important characteristic of knitted fabrics is that they frequently have two distinguishable sides (compare Figure 5A1 to 5A2, showing two sides of same fabric sheet) varying in term of texture and properties. This needs to be considered when designing the scaffold as texture may affect its blood compatibility^30^. The difference in properties of each side will often depend on the type of knitting chosen. For example, interlock stitch will provide a rough surface while a surface of velvet with closed loops will be very smooth.

**Figure 6.**
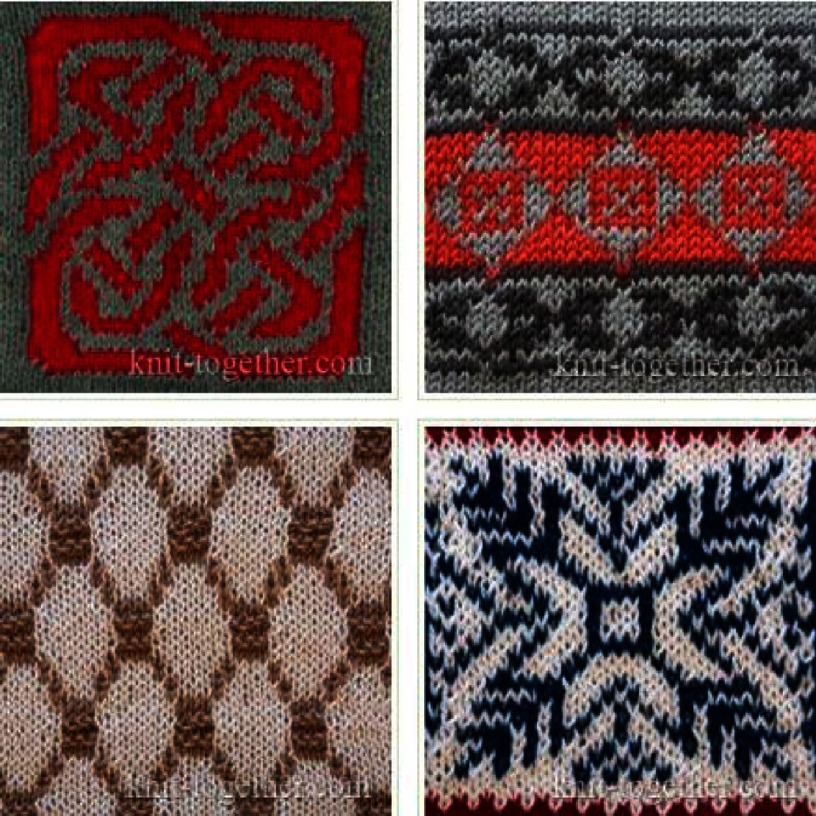
Examples of Jacquard knitting patterns. Reprinted with permission from^34^.

Another important specification of knitting is related to its scaffold coding capacities. The coding concerns the possibility of involving a specific yarn in a specific place of the construct. This provides additional precision that could be used to control the type of surface on which the cells are seeded and manipulate the cells interacting with the scaffold. Jacquard knitting (Figure 6) is one of these techniques that can create a hybrid structure consisting of several types of yarns capable of precisely controlling cell growth. In Jacquard knitting, the needles of knitter can be manipulated individually, providing virtually endless options for obtaining specific patterns.

Also in the case of Jacquard knitting, its limitation is that face and reverse sides of fabrics could be dramatically different in appearance and properties. This may be critical for leaflets, since each side should be carefully designed for cell/polymer specific interactions. This can be overcome by the lengthwise coding of a yarn prior to the knitting^32,33^.

**Figure 7.**
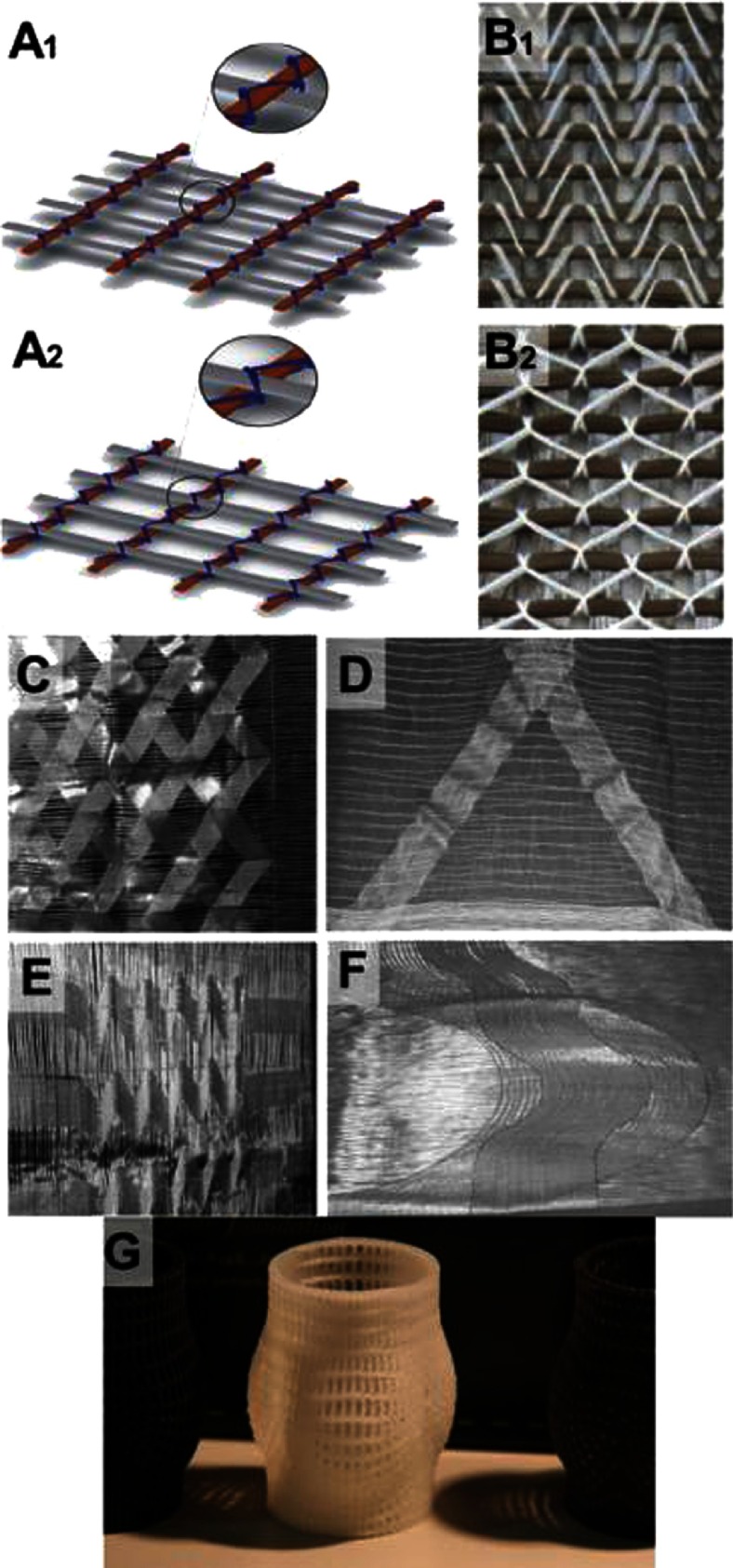
Stitch-bonded type of warp knitting. Schematic representation of face (A1) and reverse (A2) sides of composite. Stitch is blue (A). Composite made out of carbon fibers bonded with white yarn (B), face (B1) and reverse (B2) sides of composite. Examples for stitch-bonded fabrics made by using the warp offset device (C-F), two warp layers crossing (C), reinforcement for triangular perform (D), threads with reserve length (E) and segment of a circle (F). Reprinted with permission from^35^. 3D printed PCL construct that could work as a stabilization “jacket” for knitted yarns (G). Prior to forming the final shape, the yarns can be combined with pillars of the “jacket” using stitch-bonded type of warp knitting.

Another unique and important technique available in knitting is warp-stitch-bonding. It is commonly used to design composite materials, when knitted loops are used as a knot between elements. It can be particularly useful for combining the knitted structure with an enforcing “jacket” to further improve long-term dimensional stability of the construct (see Figure 7).

**Figure 8.**
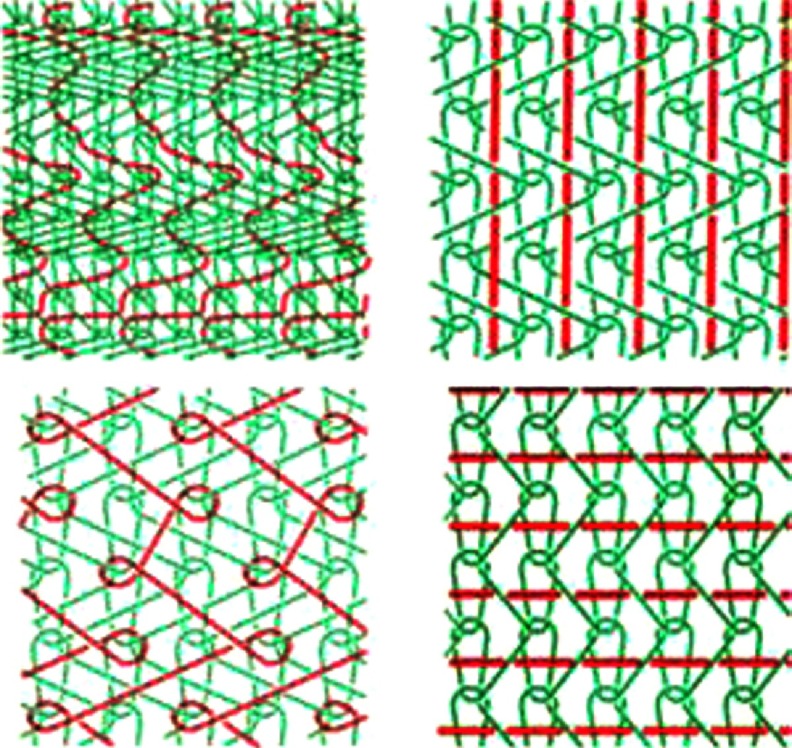
Schematic representation of four laid-in stitches. These types of stitches can be used to precisely design the direction of stiffness and elasticity. Reprinted with permission from^28^.

A striking type of knitting is a laid-in stitch, which can be used to precisely design the direction of stiffness and elasticity (see Figure 8) within the leaflet and HV. This, as a consequence, prevents deformation in a given direction. This technique could be applied to make hybrids for specific applications. For example, if the laid-in yarn is non-degradable, while the other yarn is biodegradable, the resulting scaffold could be remodeled as the ECM is being formed, while the non-degradable yarns will provide the permanent support.

**Figure 9.**
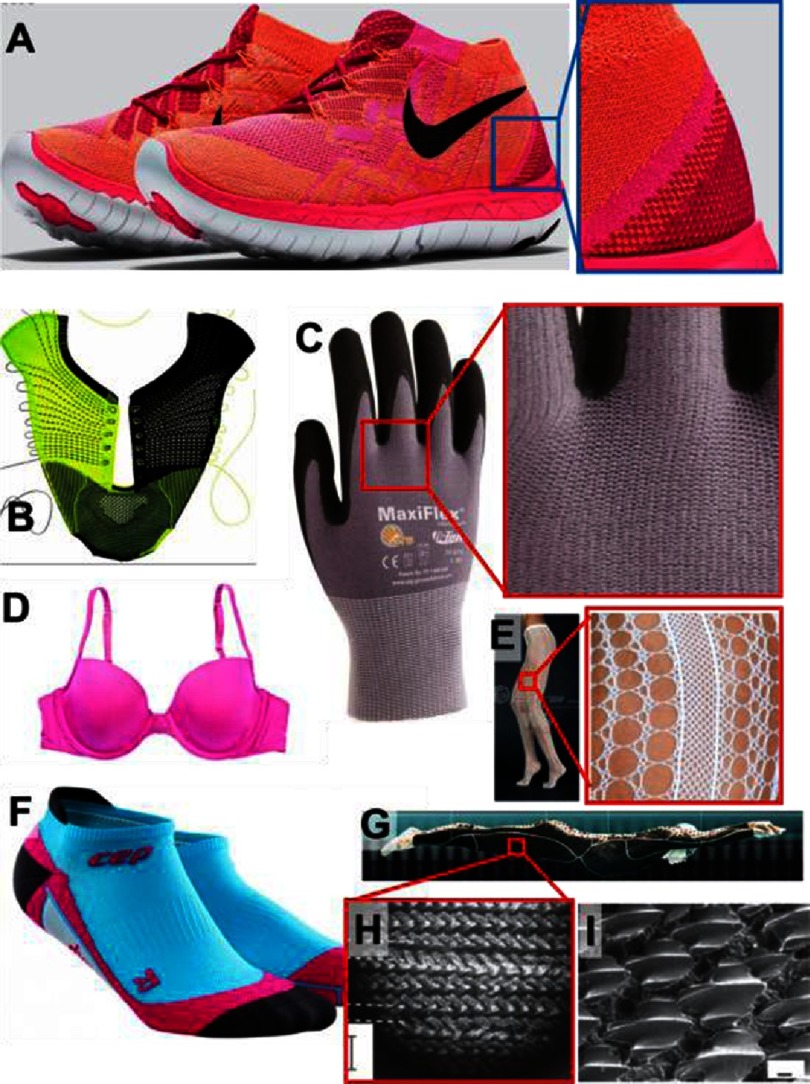
Common knitted products as inspiration for HVTE. Knitted shoes (A, B). The 3D shape shown is obtained from flat fabric, enforced with yarns (B). Knitted seamless gloves (C). Cups of bras frequently made by 3D deformation of flat knitted patches by draping on mold (D),^36^. Knitted hosiery made in one-run (E); Socks are made by combining different knitting patterns (F). In (G), a knitted swimming suit is shown inspired by sharkskin (I) to reduce resistance of water by using a specific pattern on the fabric face side (H). Images were reprinted with permission from following sources; A^37^, B^38^, C^39^, D^40^, E^41^, F^42^, G^43^, and H & I^44^.

Perhaps the most efficient way to increase awareness of the several techniques and advances in knitting technology among tissue engineers is to present several common knitted products and discuss opportunities arising from solutions specific to them. Some examples can be seen in Figure 9, which includes shoes, socks, gloves, bras, sport suites and sharkskin-inspired swimsuits.

Sports shoes (Figure 9A) are constantly exposed to dynamic mechanical stresses. Due to this, multiple areas with specific knitting patterns need to be adjusted in order to transfer the forces from the feet to the sole without damaging the body. Similar challenges can be identified in HVTE. In Figure 9B, there is an example of a flat fabric enforced with yarns to form a 3D shape; a similar concept can be used to produce a fibril network enforcing the leaflets.

Knitted seamless gloves (Figure 9C) have a complex geometry, thus the machines that are used to make them could potentially be adopted to fabricate HV geometries. Bras (Figure 9D) are made by 3D deformation of flat knitted patches draped on a mold^35^. This could be applied in HV to obtain leaflets and sinuses from a tube. Another way to obtain tubular shapes for HV could involve the technology used to obtain knitted hosiery that can have a variety of patterns (Figure 9E) in one run.

The manufacturing of socks (Figure 9F) is also interesting, since they are made by combining different knitting patterns and the method of shaping the heel could be used to make the sinuses of HV. Knitted swimming suits (Figure 9G) are inspired by sharkskin (Figure 9I) to reduce resistance of water by using a specific pattern on the fabric face side (Figure 9H). This may be particularly useful to achieve specific surface/blood interaction on a given place of HV scaffold.

After manufacturing, the fabric needs to go through finishing processes to ensure that it is usable. Finishing of knitted textile include a range of techniques that includes mechanical finishes, heat setting, chemical processes and coating^23^.

Mechanical processes include calendaring, raising and cropping. Calendaring is performed by compressing the fabric between two rolls and it provides a smooth, flattened appearance to the surface of the fabric. Raising is a type of mechanical process based on plucking the fibers from a knitted fabric in order to give a nap effect on the surface. Cropping aims to provide a smooth appearance to the fabric by shaving the surface hairs^23^. From a HVTE perspective, calendaring could be used to provide a smooth surface where blood could flow undisturbed; raising may be used to create a spongiosa-like layer on the fabric; while cropping may be used to create a specific pattern on the surface by trimming the raised fibres.

Heat setting is a process used to stabilize synthetic fibers so that they do not shrink upon heating. During the heat setting phase the amorphous and crystalline areas of the bulk yarn-polymer are rearranged, which could be used to model the construct into a specific shape. This process is frequently used for medical grafts in the final stage to shape them, and includes three steps; namely, (I) heat the fabric to within about 20–40°C of the fiber melting point, (II) hold at this temperature under the tension for approximately 20s, and (III) cool the fabric before removing tension^23^.

Chemical processing is the most important way of finishing a knitted structure for HVTE. Usually, chemical finishing involves the application of chemicals that are flame retarders and water repellants. When it comes to HV scaffold, chemical processing could be used to improve the polymer interactions with the body, such as reducing the immunological response and thrombotic effect, as well as enhancing cell specific scaffold interactions^15,16^. Prior to chemical processing, it is important to wash the fabric to remove coatings and dirt that may result from yarn production or from the knitting process.

Surface coating is currently the most prominent way of finishing medical textiles. Synthetic grafts have been coated with proteins such as collagen, fibronectin or polysaccharides including heparin to avoid graft thrombosis, mismatched compliance and anastomotic intimal hyperplasia. Depending on the proteins used, the coated grafts demonstrated better cell attachment in vitro and improved patency in vivo^45–48^.

## Three-dimensional knitting: Achievements and potential for HVTE

In principle, all of the above-discussed garments (with the exception of bras) are flat structures that only adopt a 3D geometry when stretched onto or around an object – the human body for example. An alternative to this method is 3D knitting. 3D knitting confers better manufacturing and mechanical properties since it provides a ready-made 3D structure without a need for structural support. Therefore, 3D knitting is important for HV engineering since the 3D structure of the valve must be sustained even without the pressure of blood. Similarly, the native valve is an elastic structure, which should not collapse when removed out of the heart. Thus, this branch of knitted textiles may yield the structures that are the most relevant to HV geometries^2^.

**Figure 10.**
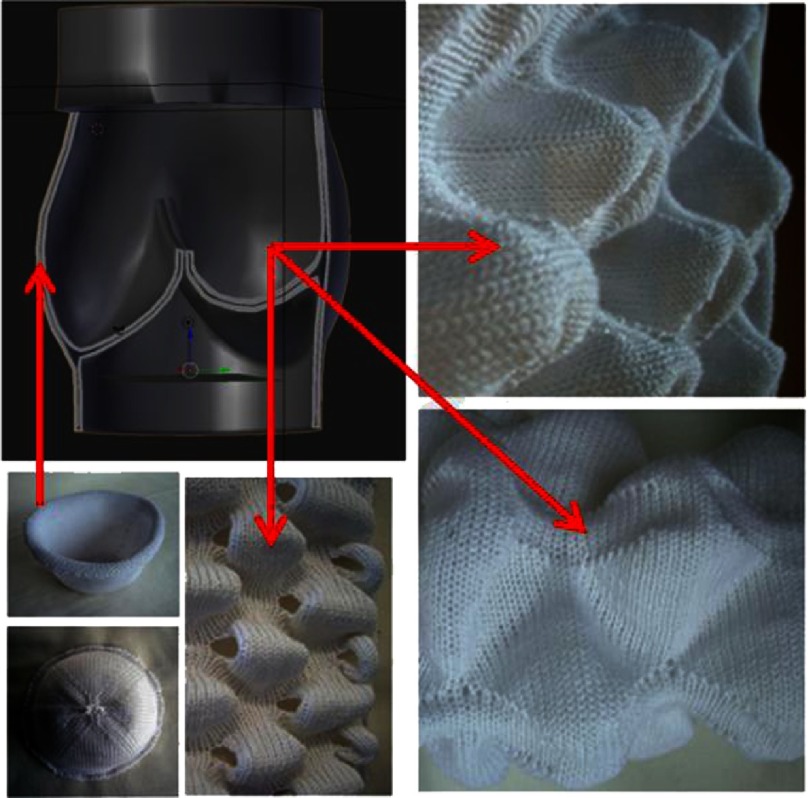
Examples of single and multiple domes, obtained by using weft knitting technique (photos) and their potential applications for constructing HVTE geometry (graphic and red arrows). Reprinted with permission from^31^.

Weft knitting, so far, has proven to be the most versatile method when it comes to 3D knitting^31^. Regarding machine type, the flatbed machines are superior over the circular knitters as they can provide multiple types of tubes and enable rib transfer, thus variations in the width and circumference of 3D forms may be achieved (Figure 10). Another important consideration is that to develop challenging geometries, the equipment needs to be controlled by cutting edge software. Important breakthrough research on modeling knitted clothing with yarn-level detail was presented by Yuksel and colleagues in 2012^49^.

Surprisingly, manual crocheting could also yield spherical seamless shapes but this process has not been yet automated, most likely due to economically insufficient market demands. Nevertheless, it is a possible alternative that needs to stay on the radar of TE.

### Knitted medical devices

Vascular grafts, cardiac nets and hernia meshes are leading the market of currently applied medical textiles. In the heart valve industry, the knitted structure is mainly used as a cover for the ring to enable suturing the valve to tissues. Nevertheless, some examples of knitted-assisted HVTE in their pre-clinical phase were reported^50–53^, which will be outlined below.

Knitted grafts are built of the filament loops that are continuously interconnected and spirally aligned around the graft circumference. Both weft and warp knits are used for graft design. Knitted structures are softer, more compliant, more flexible and easier to handle than woven structures. Weft-knitted structures have more stretch than warp knits, and therefore are inherently less dimensionally stable. Commercially available knitted implants do not vary much in their structure, thus the versatility of knitting technologies has not been fully exploited yet. The grafts used are mainly a plain-knit single layer homogenous structure, hence, more studies on making them aorta-like are in progress^54^. To the authors’ knowledge, no bioresorbable polymers have yet been used to make a clinically applicable graft. Polyethylene terephthalate has been been, so far, almost the only material used for commercially knitted grafts^55^, however there is a problem concerning its application because its mechanical behavior is significantly different to that of natural arteries^56^.

**Figure 11.**
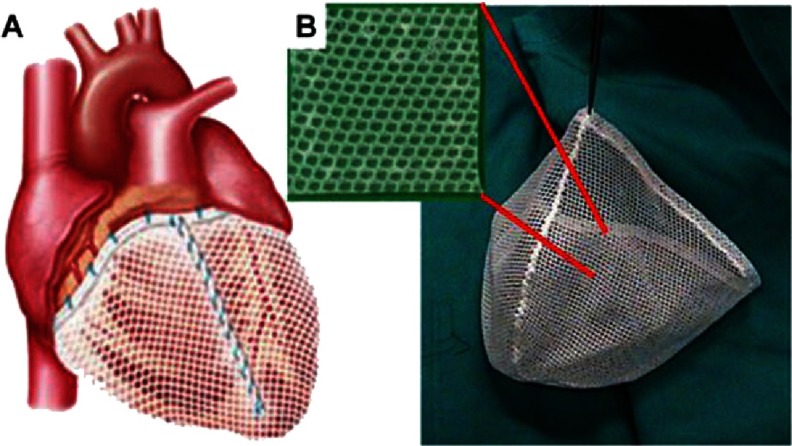
Device for cardiac support - CorCap. The appearance of the device sutured to the heart (A). Free-standing mesh prior to implantation (B). Reprinted with permission from^57^.

Another example of knitted medical textiles are nets for cardiac support, for example CorCap (Acorn Cardiovascular Inc., St. Paul, MN, USA)^57^. CorCap is a warp knitted fabric mesh implanted around the heart to provide circumferential diastolic support and limit negative left ventricular remodeling by reducing ventricular wall stress (see Figure 11).

One of the most commonly used knitted medical textiles are meshes for repair of recurrent and primary inguinal hernias. The nets are made of polypropylene or polyvinylidene fluoride (PVDF), monofilament yarns using warp knitting – with tricot and Raschel stitch (see Figure 3).

For patients with aortic dilation, a textile medical device that halts or reduces aortic root dilation has been developed and marketed by Exstent Ltd (Tewkesbury, UK). The Personalised External Aortic Root Support is a PET/Polyester textile mesh formed to the geometry of the individual patient’s aorta.

During the implementation procedure, the chest is opened, the aorta is dissected out of the adjacent structures, and the mesh is implanted around it^59^. Importantly, dissection of the aorta does not include cutting through its diameter/width, but only extracting the aorta from surrounding tissues^59^. What makes this device unique is its high level of personalization. Spatial data from magnetic resonance imaging (MRI) or computed tomography (CT) images are used to create a CAD model from which a replica of the individual’s aorta is made by 3D printing. On this mandrel, a mesh support is formed and immobilized by suturing it proximally to the left ventricular outflow tract (LVOT) and looping its distal end around the Brachiocephalic root. The implant forming process (probably textile thermo fixing) ensures the preservation of scaffold’s shape after removal from the mandrel^59^. Another very positive aspect of the ExoVasc device for personalized external aortic root support (PEARS) is integration with host tissue, which was confirmed in an animal model (Figure 13)^60^.

**Figure 12.**
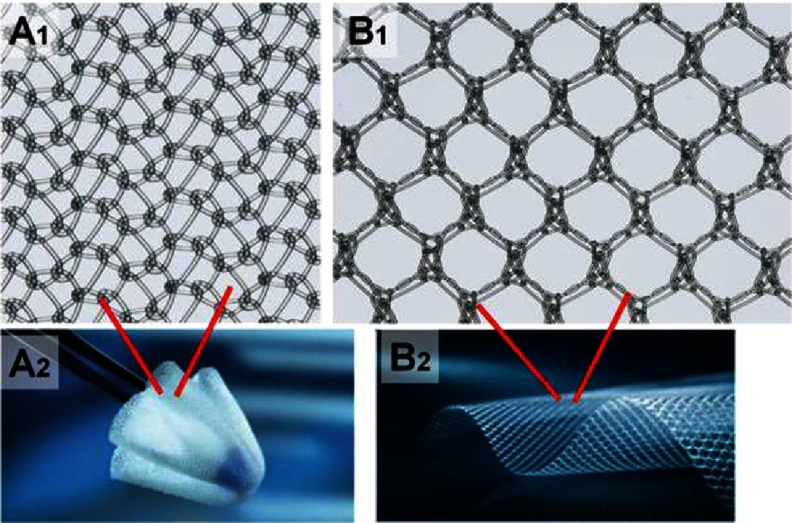
Knitted mesh for treating hernias. Polypropylene yarns knitted using Tricot (A) and Raschel (B) stitches. The mesh can adapt to the movements of the patient resulting from body motion; the pore size is 1 mm (A). The mesh is used for treating inguinal and incisional hernia; the pore size is 1,5 mm (B). Reprinted with permission from^58^.

**Figure 13.**
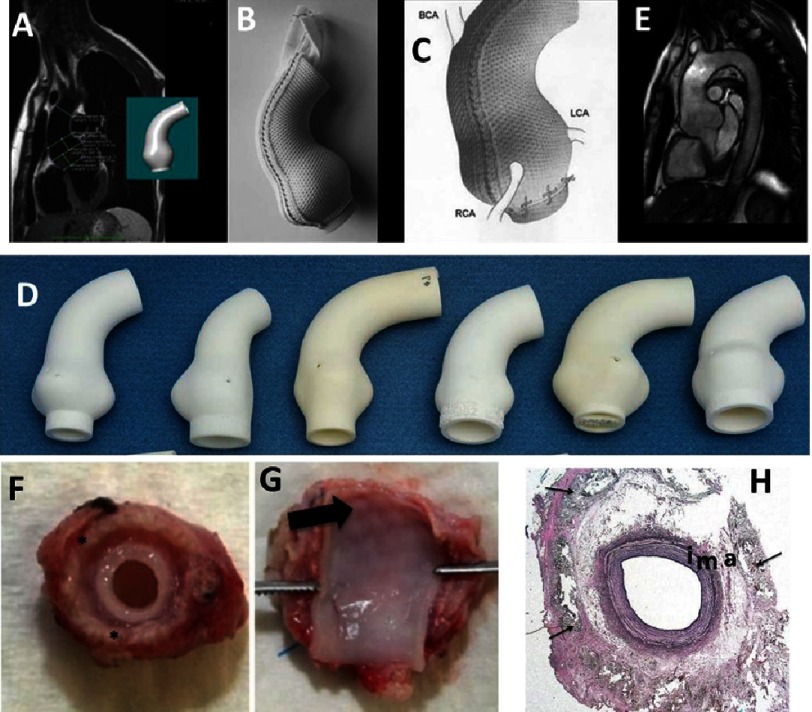
Technical aspects of personalized external aortic root support. Measurements made on the MRI and the model created from it by computer aided design (A). Soft macroporous, PET based, warp knitted, mesh sleeve (B&C) supported by the replica of the aorta made by rapid prototyping. Variety of aorta models (E). MRI scans showing aortic valve support after implementation (D). Reprinted with permission from^59^. Mesh grafted carotid artery of sheep; in some samples graft material shone through the vessel wall (arrow), (F&G). Elastica staining of the wrapped carotid artery with preserved vessel architecture (i: intima; m: media; a: adventitia) and fibrotic incorporated mesh material (arrow) in periarterial tissue (H). Reprinted with permission from^60^.

Fabric internalization is very important in regards to smart scaffold technology since the polymers must be not only integrated but also even finally bioresorbed. The authors reported no aortic or aortic-valve events in 63 treated patients, of which 30 were monitored for almost nine years after implementation. Comparing it to a standard root replacement procedure of implementing a mechanical valve, the PEARS was found to be superior as it had not caused any thromboembolic event. To support this statement authors declared further studies including a larger group of patients^59^.

One concern related to PEARS was that an aorta reinforced from outside will become thinner over time as its exposure to mechanical stimulus is restricted. In the case of PEARS, no thinning of the aorta was observed in humans^61^; in fact, animal studies showed thickening of the aorta^60^. One possible explanation could be that within the support the aorta is less exposed to diametral/tensile deflection, but wall compressive deflection compensates for this reduction. The macroporous mesh is intimately incorporated into the outer layers of the aorta, permanently fixing the fabric in place. Dilatation beyond the mesh has not been observed, but should be regarded as a significant risk - as confirmed during the animal studies (Figure 13G). Perhaps an improvement would be to use bioresorbable yarns, or/and decorating the yarns to enable recruitment and housing of specific cells migrating from the aorta.

## Knitting-assisted HVTE

Both weft and warp knitting were utilized to develop heart valves prosthesis for research purposes.

**Figure 14.**
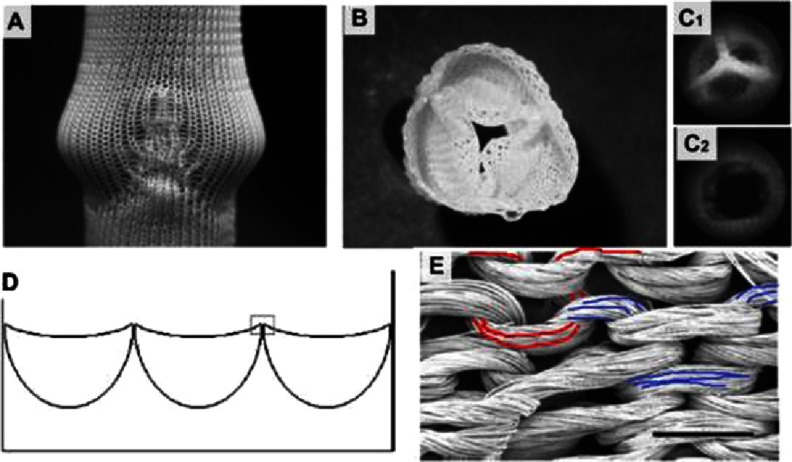
Weft knitted heart tricuspid valve. Knitted structure pulled on mold (A). Structure after fibrin formation and removal from mold (B). Static images of valve in its closed (C1) and open (C2) positions. Schema of patch design with three packets (D). The weft knitted loops of PCL yarn (E), scale bar is 1 mm. The red and blue colors mark two individual loops. Reprinted with permission from^50^.

In 2006, the Baaijens’ group reported tests performed on knitted tricuspid valves^50^. A multifilament (220 dtex f44), polycaprolactone (Mw 50,000) yarn, Grilon KE-60 (EMS Griltech, Domat, Switzerland) was used for the double-bed weft-knitting of a rectangular patch with three half-round pockets (see Figure 14). The same yarn was used to suture the patch into a tube. To clean the yarns, the scaffold was washed with water and soap prior to sterilization in ethanol. The scaffold was pulled on the valve-shaped-mold and a mixture of fibrinogen and thrombin was poured over the scaffold for gel formation. The gelation was repeated until the knit looked fully covered by fibrin. The scaffold was released from the mold and tested in pulse duplicator system, where it survived 1 million open-close cycles with no fabric rupturing.

This early work established a milestone for the textile-assisted HVTE, the importance of which is unquestionable. However, there are some drawbacks of this approach, which need to be discussed for the benefit of ongoing research. One major issue is that the knitted structure does not preserve the geometry of the valve, since fibrin-gel can be easily washed out of the yarns, as reported in Baaijens’ paper^50^. Therefore, due to the possibility of leaks, the geometry and performance of the valve may become unstable over time. This limits the possibility of hydrogels being used as a binder for the yarn, but they could still play a role as a shock absorber, lubricant and/or cell housing medium. Based on these considerations, a better use of the hydrogel in the structure of leaflet could be in the spongiosa^62^. At this location, the hydrogels should be well protected by yarns and nanofibril structures (see Figure 2) of the surrounding biopolymer. This protection needs to last long enough to enable recruited cell growth and glycosaminoglycans (GAGs) synthesis.

Baaijens et al^50^ cultured the cells on scaffold made out of the yarns; however the interaction of the cells with the yarn was not mentioned. This is unfortunate because the alignment of the cells lengthwise to the yarn and subsequent ECM orientation is crucial for designing the anisotropy of the leaflets^63,64^. The utilisation of bioresorbable polymer (polycaprolactone, PCL) for knitting was the pioneering step towards developing the concept of intelligent scaffold and successfully continued by Baaijens later^65,66^.

**Figure 15.**
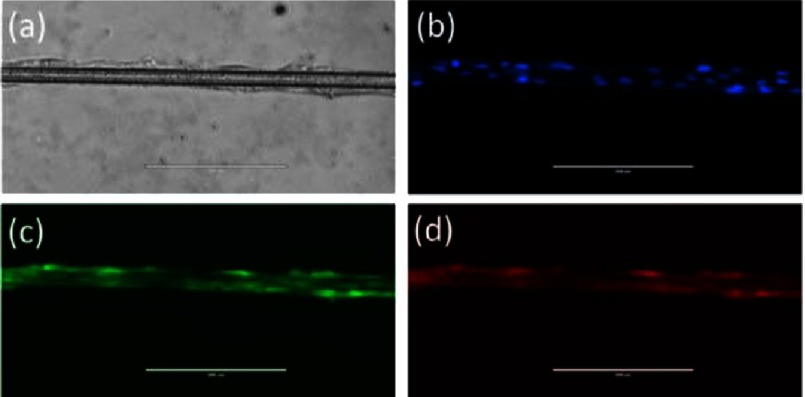
Visualization of MSCs on single filament of PCL thread using Bright Field (a), and fluorescence filters (DAPI) (b), celltracker (c), RFP (d). (Scale bars=200 µm). The cells align lengthwise to the yarn, which may potentially be useful for triggering deposition of ECM in an oriented manner, and subsequently for providing the leaflets with desired anisotropic fibres.

Based on the pioneering studies of Baaijens et al.^50^ on the PCL scaffolds, preliminary tests of human mesenchymal stem cells (MSCs) culture on PCL threads (made by the present authors) showed the longitudinal alignment of cells along the yarn (Figure 15).

Much more recent examples of applications of knitted fabrics in HVTE are reported by Mela’s group^51–53^. In these cases, the fibrin constituting the leaflets of valves is enforced using a warp-knitted tubular mesh made out of polyethylene terephthalate (PET). In all three reports, the authors evaluated biocompatibility of the construct by encapsulating the cells in the fibrin gel constituting the leaflets and quantifying the secreted ECM proteins. Other authors reported that *in vivo* implantation with fibrin-based tissue-engineered heart valves^67,68^ revealed an absence of calcification, thrombus formation, aneurysm development or stenosis. After 90 days of implantation, it was also observed that a monolayer of endothelial cells was formed^68^ which exhibits the promise of fibrin scaffolds for HVTE.

Nevertheless, it is not clear if this approach will be adaptable for the intelligent scaffold, as in the smart solutions, the cells need to be *in situ* recruited from blood and surrounding tissues.

Moreover, PET is not a bioresorbable material, thus alternative strategies need to be proposed to enable growth of valve tissue within the patient. For example, warp-beam can be set with alternating PET and PCL yarns, therefore the hybrid scaffold will be continuously enforced by PET and still able to grow after PCL degradation.

Furthermore, the leaflets are stitched to the silicone tube that simulates the valve (sinuses of Valsalva ). From an experimental point of view, this is an advantage as leaflets can be observed from the side; nevertheless, more work is needed on combining the leaflets into the implantable graft.

In summary, the solutions proposed by Mela and colleagues are promising and encouraging. To actively support this statement, we fabricated leaflets using special PET knitted fabric.

**Figure 16.**
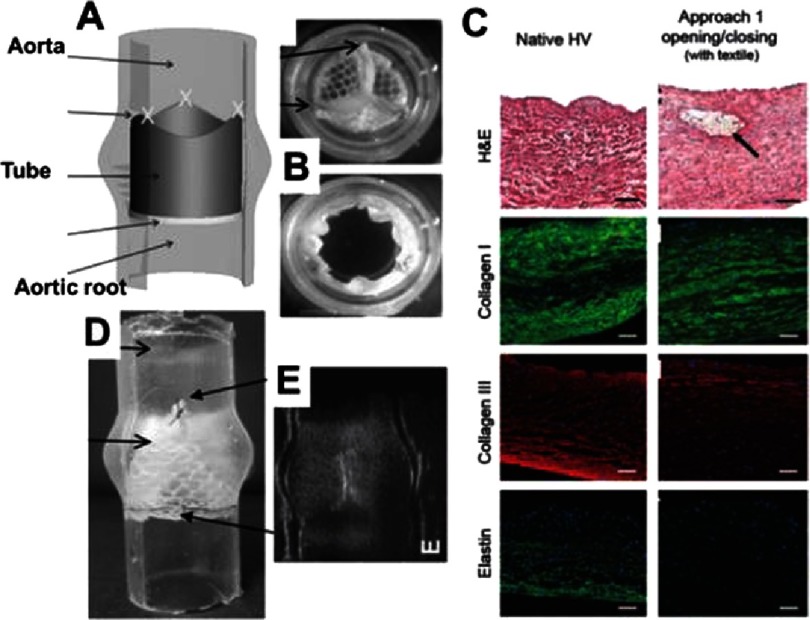
Tube-in-tube valve and its biocompatibility test. Principle of the tube-in-tube valve (A). Still images of closed and open cycles of tube-in-tube valves (B). Histological and fluorescence immunohistochemical micrographs of native ovine pulmonary valve (wall), dynamically conditioned tissue-engineered tubular valves (C). Scale bars are 100 µm. The black arrow indicates the textile structure. Sutured in a silicone tube featuring the sinuses of Valsalva (D). Ultrasound images of the tube-in-tube in closed position (E). Reprinted with permission from^51^.

**Figure 17.**
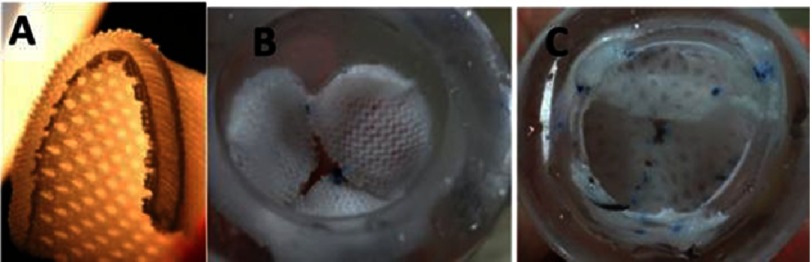
Three-dimensional weft-knitted spacer fabric made out of PET yarn, originally made for sport-garment.

## Cell colonization in knitted fabrics

Porous 3D structures are used to support and guide the ingrowth of cells into the matrix during colonization^69^. In this section, we summarize various factors affecting cell colonization in the three-dimensional environment of knitted fabrics. Cell^70,71^ material interactions (adhesion and mechanotransduction), scaffold properties such as porosity^72^, pore size, void fraction^73,74^, fiber thickness, topography and scaffold stiffness^75^ all play key roles during colonization.

## Porosity

Porosity is a measure of the open pore volume within the matrix, often called the void fraction; it is the ratio between open volume and total volume of scaffold. Knitted textiles are inherently a macroporous structure due to their loop-based nature^76^. The size of the loop can be adjusted, but the minimal size is governed by the size of needle used for knitting. Moreover, porosity, types of pores and pore interconnectivity can change dynamically upon exposure of the fabric to mechanical stress. It is acknowledged that pore size affects cell attachment, migration, depth of cellular in-growth, phenotypic expression and cell morphology^77^.

The optimal pore size range depends on the materials as well as cell types^78^; outside this range, cells fail to spread and form networks. For example, endothelial cells (ECs) are able to colonize scaffolds with pore sizes <300 µm^79–81^. The “safe pore size range” for supporting cell ingrowth for the majority of mature cells is in the range of 100–150 µm^81^. Obviously, the porosity of yarns used for knitting is an important factor to consider especially for multifilament yarns. Filaments packed in yarn will provide the fabric with pores or/and channels oriented lengthwise to the thread. One may hypothesize that these channels can be used to drive oriented cellular growth and ECM deposition. In general, it is reasonable to expect that the knitted structure will leak in contact with blood. To prevent leaking, a coating may be applied on scaffold using ECM^30^, hydrogels^51^, nanofiber meshes^82^ or polymer films. Therefore, the macroporous nature of knitted structures enhances polymer cell interaction, good contact with blood and enables cellular colonization of the structure.

## Filament/fiber thickness

An important parameter in knitted scaffolds is the microscale thickness of the individual material filaments in the yarn. The filaments in knitted structures are usually highly organized with regular repeating pore units (at fabric relaxed state). The filaments’ thickness, length, width and shape (circular, rectangular, etc.) will play the role in cellular colonization of fabrics. It was previously demonstrated that fibers (700nm in diameter) of oriented polycaprolactone nanofibers promoted phenotypic differentiation of chondrocytes compared with 2D nonporous membranes^83^. Cells maintain phenotypic shape and guided growth according to nanofiber orientation of oriented fibrous structures on which they are seeded. Scaffold fibers orientation may enhance the ECM proteins synthesis^84^. Further studies are necessary to verify if the orientation of filaments in the yarns may affect cellular colonization in a similar manner^22^.

## Filament topography

The surface characteristics of yarns constituting the knitted scaffold can be described by its topography, comprising of micro- to nano-scale material surface features. The topography of the yarn surface may influence the spreading characteristics and activity of cells^85^. The extrusion-based method of filament production provides the filaments with grooves, pores and other lengthwise homogenous topographical features. It was shown that grooves may reshape actin filaments to adjust to the new topography^86^, or inhibit cell movement and bend the cell cytoskeleton^85^. Surface roughness can significantly increase the cell migration area^87^ and improve the adhesion and growth of cells^88^ on polymer scaffold^89^. Another way of modifying the yarn surface is by the inclusion of nanoparticles or by etching the surface of the matrix^88,90^.

## Stiffness of knitted structures

The resistance of the material to deformation (stiffness) influences cellular activity^91,92^. Cells show reduced spreading when soluble adhesive ligands are not strongly attached to the surface^93,94^, as scaffolds are unable to withstand cell contractile forces^92^. The stiffness of the knitted construct can be increased by heat-setting the fabric^23^; this needs to be performed carefully since an elevated temperature may permanently melt and join filaments of the knitted yarn together and change porosity. Raising^23^ is another finishing technique that may be used for changing the structure and properties of the material, such as enhancing cell /polymer interaction on a given side of the fabric.

## Mechanotransduction in knitted structures

Since knitted structures are more elastic than woven ones, the seeded cells would sense more stretch and deformation when the knitted fibres are subjected to stretching. Studies have shown that both mechanical stresses^95^ and hydrodynamic stresses^96^ affect cell colonization. Shear stress initiates multiple signal transduction cascades leading to functional changes in endothelial cells^23^. Endothelial cells grown in a perfusion reactor have been shown to align in the direction of flow^97^. Mechanical forces may unfold selected protein domains, thus providing access to a different set of binding sites or trigger opening or closing of ion channels^98^.

Cells experience local shear stress rates when flow through the scaffold microarchitecture occurs. The scaffold architecture controls the transport of nutrients within the samples. However, the dynamically changing geometries of knitted structures may result in flow irregularities, extracellular matrix washout or local hypoxia^98^. The presence of flow within a reactor also affects the production of ECM components. Therefore, the flow that the scaffold is subjected to in a bioreactor must be closely regulated to control cell seeding and ECM production.

## Cell adhesion on the yarn surface

Another aspect that influences the interaction of the knitted scaffold with the surrounding environment is the yarn surface. Cellular adhesion, proliferation and differentiation can be enhanced by anchoring to the scaffold surface specific cellular binding sites^99^ or incorporating into the scaffold growth factors^100,101^.

The knitted structures are often used in a hybrid format with hydrogel; namely, collagens, glycosaminoglycans (GAGs)^102,103^, alginates or fibrin gel^50–53^. The hydrogel is used to host the cells and enable their proliferation but it can also be loaded with drugs or agents responsible for scaffold remodeling. One elegant example of such use of hydrogel was reported by Baaijens, who loaded hydrogels with fast-releasing monocyte chemoattractant protein-1 (MCP-1). MCP-1 released from the gel triggered inflammation-mediated tissue remodeling^104^.

Another vital method of modifying the properties of yarn surfaces is culturing cells on them. For example, mesenchymal stromal cells (MSC), when seeded on a scaffold secreted a cocktail of trophic factors including MCP-1, which initiated a cascade of immune responses culminating with tissue remodeling on scaffold^105^. This strategy has not been applied yet for knitted fabrics but there are no obvious obstacles to do so.

The overall efficiency of cellular colonization of knitted fabric is related to structure and degradation rate of polymers used as yarns. For example, biodegradability decreases with increase in molecular weight^106^. A similar decrease of biodegradability is associated with denser structure of the crystalline sample, since it would be more impermeable to the enzymatic attack^107^.

Importantly, as reviewed elsewhere^69^, the structural, biological and degradation properties of the EC matrix also are key factors regulating colonization.

## Basic methods for characterizing knitted scaffold

The following section will focus on specific requirements for tissue-engineered constructs that textile specialists need to understand. This is important because obstacles encountered during the scaffold preparation need to be addressed in a manner that would not affect the requirements for its final application. For example, for textile engineers the obvious way of providing 3D geometry to the fabric is to seam the material. However, from the perspective of tissue engineers, stitches in the construct may be beneficial (as a local enforcement), but in other occasions, stitching may need to be avoided, especially if homogeneity of the fabric is desired.

## Standardized methods for HV scaffolds characterization

The critical parameters of a scaffold and its characteristics are precisely described in the official regulations of International Organization for Standardization (ISO). For example, ISO 5840, entitled *Cardiovascular implants – Cardiac valve prostheses*, consists of three parts: General requirements^108^, Surgically implanted heart valve substitutes^109^ and Heart valve substitutes implanted by transcatheter techniques^110^.

According to ISO 5840, the heart valve substitute should meet following basic conditions:

• allow forward blood flow with acceptably small pressure difference,
• prevent retrograde flow with acceptably small regurgitation,
• be biocompatible,
• be resistant to haemolysis,
• resists embolization and thrombus formation,
• compatible with *in vivo* diagnostic techniques,
• deliverable and implantable in the target population,
• remain fixed once placed, have acceptable noise level,
• have reproducible function, maintain its functionality for a reasonable lifetime,
• consistent with its generic class and also maintain its functionality and sterility for a reasonable shelf life prior to implantation.

These regulations concern all the components of the heart valve substitute.

## Fluid mechanical performance

Hydrodynamic testing is used to ascertain information on the fluid mechanical properties within the valve. The testing provides indicators of valve performance in terms of load to the heart, and potential for blood stasis and damage, which is important from the point of view of risk assessment. The tests should include steady flow testing, pulsatile flow testing, and steady leakage flow testing. Where applicable, testing should be performed using a test fluid of isotonic saline, blood or a blood-equivalent fluid whose physical properties (e.g. specific gravity, viscosity at working temperatures) are appropriate to the test being performed. The risk assessment shall play a role in the choice of the test fluid.

## Valve lifetime estimation

The durability assessment allows an estimation of the proper function of the scaffold over its lifetime. The testing should prove that the heart valve substitute (in case of a flexible one) will remain functional for 200 million cycles. During the durability testing, the defined target peak differential pressure across the closed valve shall be maintained for 95% or more of all the test cycles. The testing should also be conducted under in vivo conditions for fatigue performance of the heart valve substitute and all its components to determine the structural life time of the material.

## Preclinical tests for heart valves

Preclinical evaluation *in vivo* should be focused on heamodynamic performance of the heart valve assessed via *in vitro* testing, surgical handling of the scaffold and its accessories, and various biological reactions of the material with the tissue, mainly:

• healing characteristics (pannus formation, tissue overgrowth)
• haemolysis
• thrombus formation
• embolization
• foreign body reaction (inflammation, rejection)
• calcification (flexible valves)
• acoustic characteristics (rigid valves), if manufacturer claims are made on this issue
• structural deterioration and/or non-structural dysfunction
• cavitation

## Biocompatibility of materials and components in the heart valve

Biocompatibility of materials and components used in heart valve substitutes should be determined in accordance to guidelines described in ISO 10993-1. During the hazard identification stage of a biological safety evaluation, the information obtained should be sufficient to allow identification of potential toxicological hazards and the potential for effects on relevant haematological characteristics. For heart valve substitutes using animal tissue or their derivatives, the risk associated with the use of these materials should be evaluated in accordance with EN 12442-1/2/3:2008.

## Other properties of materials and components in the heart valve

From the point of view of material performance and risk assessment, it is crucial to know the basic properties of materials after processing and modifications. The evaluation should include bulk, surface, chemical, and mechanical properties of material. All measurements should be performed on materials or components as they would be found in the finished product. This includes all subsequent treatments after the fabrication.

The evaluation of textile scaffold bulk properties includes: identification of materials in scope of the chemical composition and purity, density, porosity as well as permeability, microstructure e.g. defects formation, glass transition temperature, melt index, melting point, hydraulic expansion and biostability in physiological fluids.

For the modified scaffold, important assessment methods should include:

• surface chemical composition
• surface topology/roughness
• surface charge and charge density
• critical surface tension

All the measurements should be performed *after* all treatments e.g. sterilization, as these may physically affect the surface properties of the material.

Mechanical and chemical engineering properties allow the evaluation of the performance of the heart valve scaffold in the intended site and also provides information on the possible risks resulting from scaffold processing and materials used. These properties include: wear resistance, friction coefficient, peel strength, flexural and compressive strength, tensile strength, tensile strain to failure, strain energy to failure, residual stress, stress relaxation, fracture toughness, crack growth velocity, fatigue life, potential stress corrosion, tear strength, Young’s modulus, Poisson ratio, dynamic moduli including storage and loss moduli.

Nevertheless, in the early stages of scientific development and proof of concept communications, the spectrum of analysis should be narrower, as its aims are to enable scientific discussion, open ideas for critique and make subsequent improvements.

Tissue engineering cannot be dissociated from relevant clinical standards and related safety issues. Therefore, it is especially important to develop a good understanding of the limitations that need to be addressed^111^. To illustrate this with an example, when it comes to knitting the HV scaffold with bioresorbable PCL yarns, the following issue should be noted. PCL has a low melting temperature, therefore devices made from it cannot be autoclaved. Also, sterilization via high-energy irradiation may rearrange the chemical bonds in PCL. This will affect biocompatibility and degradation rates^112^. Moreover, immediately after implementation, PCL starts to degrade. It is reasonable to expect quicker degradation in places subjected to higher dynamic mechanical stress. Perhaps at these locations, the scaffold will need to be enforced by less fragile polymers. Regions of the scaffold unoccupied by cells may degrade and yield short fibrils that will circulate in blood. It is not clear if, in that form, the PCL would degrade quickly enough to avoid a build up of plaques inside the veins.

## Basic mechanical tests of HV scaffolds

There are several methods to mechanically characterize scaffolds for HVTE. Usually, the first tests involve tensile testing. Tensile testing is mainly performed in a uniaxial machine, but it could also be done biaxially. This test gives a measure of the sample’s elastic modulus (stiffness, E), ultimate tensile strength (UTS), strain-to-failure (ε_f_), yield stress and yield strain. Several studies^113–115^ determined average values for these properties in native valves, which are displayed in Table 2, where *circ* is the circumferential direction and *rad* is the radial. It is important to remember that the leaflets contain collagen fibers that are not initially straight. As the tensile test progresses, the fibers will be stretched until they are straight, which will require higher stresses. This will translate to an initial region in the curve where the modulus is low, which will develop to an elastic region with higher modulus, when the collagen fibers are straight. The values of moduli displayed in Table 2 correspond to the region where the fibers are already straightened.

**Table 2:** Typical values obtained from tensile testing for native valves.

Sample	E_circ_ (MPa)	E_rad_ (MPa)	E_circ_/E_rad_	UTS circ (MPa)	UTS rad (MPa)	ε_f_ circ (%)	ε_f_ rad (%)
Sheep AV^114^	3.84	∼0.2	∼19,2	∼2	∼1.5	∼20	∼50
Fresh porcine AV leaflets^117^	54.6 ± 7.4	7.82 ± 0.58	∼7	6.25 ± 0.9	1.18 ± 0.09	30.8 ± 3.2	62.0 ± 3.5
Porcine AV leaflets^118^	28 ± 10	1.33 ± 0.42	∼21				
Porcine AV^116^	3.35	1.09	3.2				
Human AV^116^	3.52	2.27	∼1.6				
Porcine ventricularis	7.41 × 10^−3^	3.68 × 10^−3^	∼2				
Porcine fibrosa^119^	13.02 × 10^−3^	4.65 × 10^−3^	∼2.8				

Studies on knitted scaffolds have to be able to achieve similar values to the native valve in tensile testing. Table 2 shows a great range in the values of elastic modulus for heart valves. This could be explained due to species variability, however even when comparing studies in porcine valves, the highest value for circumferential elastic modulus is 20x higher than the lower value^116,117^.

Lee et al. used strips of 5 mm (circumferential) and 8 mm (radial), explaining that strips with less than 4 mm and 6 mm in width, respectively, did not produce a stable response after preconditioning with cyclic loading and eventually failed, therefore they could not be representative of the *in vivo* structure^117^. This should be taken into account when undertaking mechanical tests, since the preconditioning must be appropriate. Sauren et al. found that it is necessary not to exceed the first elongation in subsequent cycles to achieve the required preconditioning^118^. It was also found that aortic valves are preconditioned after a maximum series of ten cycles.

Another important mechanical test, especially for knitted scaffolds, is burst strength. This test exposes the sample to increasing pressures until structural failure occurs, a moment characterized by a loss in pressure. Table 3 displays several values for the burst strength of the scaffolds.

TexMi is a mitral valve scaffold made with fibrin in a knitted PET structure^53^. The knitted structure doubles the strength of the fibrin scaffold to 90 mmHg. This increase is greatly enhanced by the TexMi design to a value of ∼555 mmHg. However, this value is still significantly lower (only 10%) than the one obtained for ovine mitral valves. In the tube-in-stent fibrin-based PET knitted scaffold made by Moreira et al.^53^, a burst pressure of 146.1 ± 46.0 mmHg was measured. After the cultivation with endothelial cells for 21 days, the valves were crimped from 22 mm to 8 mm and kept in the crimped state for 20 min before returning to the original size. The test was performed again and the burst pressure before crimping was increased to 321.1 ± 30.9 mmHg.

After crimping, the value did not significantly change. This is an important consideration, because during the implantation of the valve, crimping will occur, and this has been shown to damage the collagen and endothelial layer^120^. The similar results for burst strength before and after crimping obtained by Moreira et al.^53^ can be explained by the reduced time in the crimped condition. Therefore, when designing a knitted scaffold, the effect and duration of crimping must be taken into consideration to see if the damage to the endothelial layer will significantly decrease the mechanical properties.

**Table 3:** Values for burst strength of different scaffolds.

Material	Burst strength (mmHg)
Fibrin without textile reinforcement^51^	44.0 ± 18.5
Fibrin with textile reinforcement^51^	90.7 ± 14.0
TexMi valves^51^	554.7 ± 92.2
Native ovine mitral valve^51^	5272.3 ± 538.0
Tube-in-stent PET textile with fibrin^53^	146.1 ± 46.0
Tube-in-stent PET textile with fibrin after cultivation^53^	321.1 ± 30.9
Tube-in-stent PET textile with fibrin after cultivation and crimping^53^	334.7 ± 34.3

Finally, the mean pressure gradient and regurgitation of the knitted scaffolds must also be evaluated. The measurements of the pressure must be performed upstream and downstream of the valve at aortic conditions: 80-120 mmHg aortic pressure (100 mmHg mean), 70 bpm frequency, 5L/min cardiac output in a pulsatile flow^53^. Commercially available transcatheter aortic valve implantation (TAVI) systems have a mean gradient of 9.3 ± 4.5 mmHg^121^. A similar value was obtained with the tube-in-stent knitted PET with fibrin^53^ that had a mean pressure gradient of 7.3 ± 1.5 mmHg before and 6.8 ± 1.7 mmHg after crimping, values that were statistically not significantly different. The regurgitation was 15.1 ± 2.5 % before and 15.3 ± 3.6% after crimping, below the 20% limit defined by ISO 5840:2012 for a 23 mm valve replacement. In the knitted fibrin-covered PCL scaffold described by Lieshout^50^, the regurgitation was 39 ± 3%, due to fibrin-related leakage. This value is unacceptable, therefore the method and materials used to create this scaffold need to be revisited.

## Basic biological tests of HV scaffolds

In addition to assessing mechanical properties, the ability of the scaffold to attract and grow cells that will produce the ECM matrix must also be studied. This includes, after culture with the desired cells, histology studies, immunohistochemistry, ECM content assays, and microscopy techniques such as scanning electron microscopy (SEM).

Endothelial cells, smooth muscle cells (SMC) and fibroblasts are some of the cells used for culture of scaffold for HV. The source of the cells to be isolated is an important factor. It has been shown that tissue engineered vascular constructs made with arterial cells developed a significant synthesis of elastin that did not occur in the constructs made with venous cells^122^. Elastin plays an important role in the elastic deformation of valves, and in HVTE elastin is hardly formed *in vitro*^123^. However, the TexMi scaffold^51^ showed a significant synthesis of elastin in the stress lines with cells from ovine umbilical veins, while cells from ovine umbilical arteries did not produce elastin. Therefore, the cell source is a meaningful variable in culture of scaffolds for HVTE. Cell culture ideally should be performed in dynamic cultivation, in a bioreactor for conditioning and to obtain the proper phenotype. After 21 days, ECM production is expected to occur. The protocol varies, for example, TexMi^51^ uses 5 days in static cultivation, 6 days at 30 bpm, 6 days at 40 bpm and 2 days at 60 bpm, while tube-in-stent^53^ uses 7 days in static cultivation, then 14 days in dynamic conditions that varied from 20–35 bpm.

For the histology, the scaffold should be fixed, embedded and sectioned after culture. Morphology of the tissue can be analyzed by using Hematoxylin and Eosin (H&E) stain, and tissue development can be analyzed by Gomori’s Trichrome stain. H&E should show a homogeneous distribution of cells across the scaffold’s thickness and Gomori’s Trichrome, in the context of HVTE, should show the presence of collagen fibers.

In immunohistochemistry, the tissue should be incubated with antibodies that stain against α-smooth muscle actin (α-SMA), collagen I, collagen III, elastin, GAGs, CD31 (endothelial cell marker) and fibrinogen. Culture on TexMi^51^ produced type I and III collagen longitudinally across the thickness of the scaffold. In the native valve, collagen is the main ECM protein. Collagen content can be assessed by a hydroxyproline assay^124^. SEM should be used to analyze if a homogeneous layer of cells was obtained. In the case of crimping^53^, SEM will be particularly useful to determine the amount of damage to the endothelial layer.

## Basic tests of the knitted fabrics

From the perspective of knitted textile characterization, the list of pivotal parameters and measurements includes; thickness, surface mass, burst strength and identification of fibers in textiles.

Thickness should be evaluated according to ISO 5084-1999 *Textiles - Determination of Thickness of Textiles and Textile Products* and surface mass should be evaluated according to PN-EN 12127:2000. Reduction in the above-mentioned parameters improves the long-term biocompatibility of scaffolds^125^. Those parameters are crucial as they influence material elasticity. Scaffolds need to be elastic and conformable, on the other hand should not be too flexible as this could cause the knitted fabric to curl.

To alter the properties of fibres, many producers use chemical modifiers during the fibre forming process. Hence characterization and analysis of the chemical constitution of a fibre are essential to secure safe usage of scaffolds. It is important to determine all types of fibers present in a sample of textile material of unknown composition. The standard outlining analytic methods used to determine chemical composition of textile material is ASTM D276 “Standard Test Methods for Identification of Fibers in Textiles”. This standard only allows identification of the generic types of fibers present in a sample of textile material of unknown composition.

Since knitted woven fabrics contain oil, lubricants and delustrants, an integral part of the production process, but unacceptable in medical products, the appropriate surface cleaning method should be applied. Van Lieshout et al.^50^ proposed a simple method of removing contaminants from yarn. To dispose of the antistatic build-up layer of oils, the scaffold sample was washed in soap water for 24 hours, flushed with water extensively, and shaken in 70% alcohol overnight to obtain a sterile aortic valvular scaffold.

Due to the fact that 3 to 5 litres of blood is pumped through the valve during the cardiac cycle in each minute^126^, the scaffold should have sufficient mechanical properties to work in such conditions. Burst strength testing is a common method to characterize the mechanical behavior of scaffolds by determining the pressure at which structural failure occurs. It should be assessed according to ISO 13938-2:2002 “Textiles - Bursting properties of fabrics - Part 2: Pneumatic method for determination of bursting strength and bursting distension” Standard. It should be noted that this test must be performed in two states, dry and wet (in physiological fluids). The strength can be different in both states. Additionally tensile properties of knitted fabrics shall be evaluated according to ISO 13934-1:2013 “Textiles - Tensile properties of fabrics - Part 1: Determination of maximum force and elongation at maximum force using the strip method”. The method specifies the determination of the maximum force and elongation at maximum force of test specimens in equilibrium with the standard atmosphere for testing and of test specimens in the wet state. Moreover the knitted scaffold should open and close properly. In^127^ authors showed that the knitted scaffold is stronger than the spun scaffold which remained intact under physiological loading, whereas the spun scaffold is not.

Because scaffolds will have to be in contact with blood and human fluid, the scaffold should have pH similar to pH of blood (ISO 3071:2007 “Textiles - Determination of pH of aqueous extract”).

## Conclusions and future directions

Knitted structures appear to be a suitable candidate for HVTE application owing to their inherent design and structural flexibility. In addition, it is possible to design anisotropic elasticity in knitted structures, which could eventually be tailored to match the one present in the native valve. Some of the latest developments in knitted meshes use biological coatings to improve the tissue growth. This also can help to model the structure to match the anatomy of the implant site^30^. To make the concept of intelligent scaffold a reality, much more work needs to be done on enhancing yarn-cell specific interactions. This involves two distinct directions that will be explored in the future: modifications of the final construct and modification of yarns prior to knitting. Some promising examples of cell specific polymer surface modifications have already been reported and include enhancing the polymers with antibodies, peptides, aptamers and enzymes. The textile-based smart scaffold requires a combined input from the advanced biomechanics and textile design together in order to realize the full clinical potential of textile HV prosthesis.

**Figure 18.**
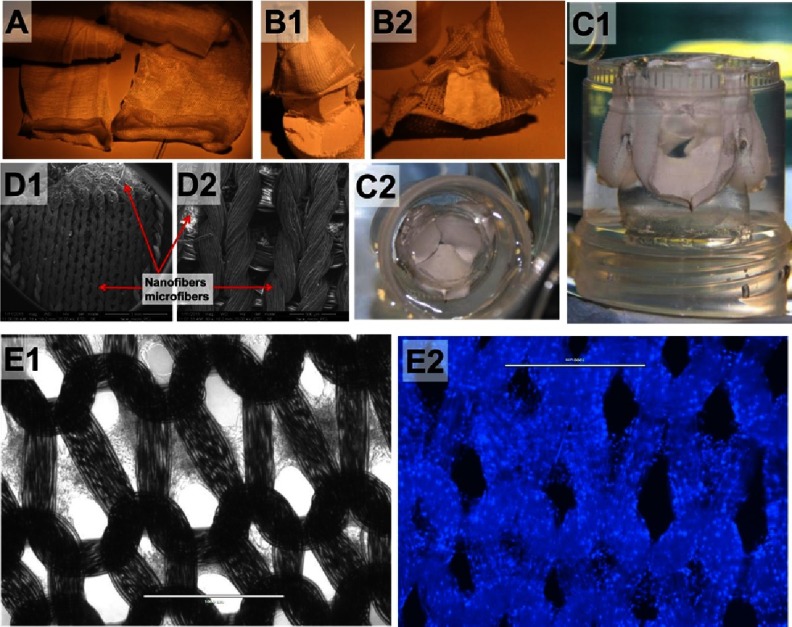
Weft knitted PCL yarns (A). Leaflet shaped using PCL fabric and PCL anisotropic nanofibril nonwoven sheets (B1), view from aortic side (B2). SEM of composite of knitted PCL microfibers and non woven sheet of PCL nanofibers (D). Knitted fabric shaped in leaflets cast in PDMS mold, side view (C1) and aortic side view (C2). The interior of mold is shaped in valve geometry. Performance of valve was roughly illustrated in water bath, see video at^128^. Adipose derived stem cells growing on knitted loops of PCL yarns (E), Bright Field image (E1), and visualization of cells’ nuclei, after staining with DAPI, image taken with DAPI filter (E2). Scale bars are 1 mm.

The general drawback of knitting is that it inherently provides a highly porous structure that can leak upon exposure to fluids. This can be, to some extent, overcome by applying hydrogels (fibrin, HA, alginate or others^62^) as a sealant. A very attractive way of sealing the knitted structure is combining it with woven or non-woven fabrics. The example in Figure 18A–18D, shows a knitted leaflet on which the anisotropic sheet of PCL nanofibres was glued. A further important potential of knitted structures is that the loops architecture can guide the growth of cells and ECM secreted by the cells (Figure 18E). More research is needed to confirm if, after yarn degradation, the structure will preserve the “imprinted” elastic properties. In fact, this is important not only for HVTE but for cardiac regeneration in general. In myocardial infarction, damaged myocardium is replaced by scar tissue that is less elastic than normal myocardium. Although the scar tissue consists of viable cells capable of producing isotropic fibril ECM proteins, it is very stiff. A bioactive knitted structure is then an appealing option that may yield an elastic living construct made out of aligned ECM proteins from the cells that have colonized that yarn. To summarize, the future is bright and even the sky is not a limit (knitted yarns are also used as an element of composites use in astronauts’ space suits).

This paper also reviewed the design aspect of knitted textiles intended for HVTE. The three dimensional structure of leaflets and their unique mechanical properties (non-linearity, anisotropy, compliance) have been widely researched and reported^1,2^. However, these features remain widely neglected while designing the scaffolds for HV replacement. Currently available HV substitutes cannot remodel and be replaced by endogenously recruited cells^129^. Moreover, their structural geometry is often not analogous to native HV. This difference can later lead to clinical complications arising from behavioural mismatch at the implant anastomosis. These observations ultimately raise the importance of understanding the structure and biomechanics of an HV before adapting a textile structure from its conventional application area to a biological environment consisting of complex structure and functions such as the aortic valve. A smart valve scaffold with structural characteristics that closely resemble a native valve will present less complications which, in turn, translates to a longer-lasting durable implant.
